# Oleosome-Associated Protein of the Oleaginous Diatom *Fistulifera solaris* Contains an Endoplasmic Reticulum-Targeting Signal Sequence

**DOI:** 10.3390/md12073892

**Published:** 2014-06-30

**Authors:** Yoshiaki Maeda, Yoshihiko Sunaga, Tomoko Yoshino, Tsuyoshi Tanaka

**Affiliations:** 1 Division of Biotechnology and Life Science, Institute of Engineering, Tokyo University of Agriculture and Technology, 2-24-16, Naka-cho, Koganei, Tokyo 184-8588, Japan; E-Mails: y_maeda@cc.tuat.ac.jp (Y.M.); okihihsoy1985@gmail.com (Y.S.); y-tomoko@cc.tuat.ac.jp (T.Y.); 2 Core Research for Evolutionary Science and Technology (CREST), Japan Science and Technology Agency (JST), 5, Sanbancho, Chiyoda-ku, Tokyo 102-0075, Japan

**Keywords:** signal sequence, endoplasmic reticulum, diatom oleosome-associated protein, *Fistulifera solaris* JPCC DA0580, marine oleaginous diatom

## Abstract

Microalgae tend to accumulate lipids as an energy storage material in the specific organelle, oleosomes. Current studies have demonstrated that lipids derived from microalgal oleosomes are a promising source of biofuels, while the oleosome formation mechanism has not been fully elucidated. Oleosome-associated proteins have been identified from several microalgae to elucidate the fundamental mechanisms of oleosome formation, although understanding their functions is still in infancy. Recently, we discovered a diatom-oleosome-associated-protein 1 (DOAP1) from the oleaginous diatom, *Fistulifera solaris* JPCC DA0580. The DOAP1 sequence implied that this protein might be transported into the endoplasmic reticulum (ER) due to the signal sequence. To ensure this, we fused the signal sequence to green fluorescence protein. The fusion protein distributed around the chloroplast as like a meshwork membrane structure, indicating the ER localization. This result suggests that DOAP1 could firstly localize at the ER, then move to the oleosomes. This study also demonstrated that the DOAP1 signal sequence allowed recombinant proteins to be specifically expressed in the ER of the oleaginous diatom. It would be a useful technique for engineering the lipid synthesis pathways existing in the ER, and finally controlling the biofuel quality.

## 1. Introduction

With an increased demand for a sustainable energy supply, biofuel production has attracted much attention. Microalgal biodiesel production has been researched to meet such demand due to its advantageous features (e.g., global carbon dioxide fixation, no competition for food, much higher biomass yield than higher plants, and oil accumulation at a high level inside the cells) [1]. Several oleaginous microalgae can accumulate triacylglycerol (TAG) in high level as a form of the oleosome (also known as oil body), and such promising oil producers have been intensively studied to understand the TAG biosynthesis [2,3,4,5,6].

A current trend in this field is genome and transcriptome analyses to determine the active synthesis pathways for fatty acids and TAG in the target oil-producing organisms [7,8,9,10,11], while proteome analysis has also been launched to identify the proteins closely attached around the oleosomes. The proteomic approach is expected to identify the novel protein machineries directly participating in the oleosome formation, which conventional pathway analysis can hardly address. It leads to the elucidation of the biological mechanism for oleosome development, and can provide promising targets of genetic engineering for the purpose of oil production improvements. However, the oleosome-associated proteins have been studied in only a few microalgae [12,13,14,15,16,17,18,19].

Among such rare examples, we have focused on *Fistulifera solaris* JPCC DA0580, an oleaginous marine diatom screened from our marine microalgal culture collection [17]. Beneficial features of this strain for practical biodiesel production include a high growth rate, high lipid content (up to 60%, *w*/*w*), a low unsaturation degree of the accumulated lipids, as well as ease of mass cultivation [20,21,22,23]. Recently, the proteome analysis for this diatom identified two oleosome-associated proteins, G12504 and G16188 (formerly g4301 and g6574, respectively) [17]. The GFP-fusion experiment demonstrated that G16118 (tentative potassium channel) showed a broad subcellular distribution including the oleosome. In contrast, G12504 (containing a quinonprotein alcohol dehydrogenase-like domain) exhibited a strict localization only on the oleosomes, implying that this protein could play a role for oleosome formation, and contain a specific signal sequence directing the proteins onto the oleosomes. This notion led us to further research the sequence features of this diatom-oleosome-associated protein, G12504 (referred to as DOAP1 in this study) in detail, and encouraged us to estimate the routing mechanism of this protein into oleosome-targeting. Particularly we focused on the *N*-terminal signal sequence which primarily governs the localization of the nuclear-encoded polypeptides within the cell organelles [24].

In this study, we carefully determined the *N*-terminal signal sequence of DOAP1, and fused it with GFP in order to examine the initial localization of this oleosome-associated protein. Fluorescent microscopy of the fusion protein revealed that the signal sequence of DOAP1 could transport proteins into endoplasmic reticulum (ER) of *F. solaris*, implying the initial localization of DOAP1 at the ER and subsequent transportation to the oleosomes. Additionally, this study also means the success in specific expression of the recombinant protein into the oleaginous microalgal ER where important lipid synthesis reactions occur [25]. It would be useful for future metabolic engineering for improvement of biofuel quality.

## 2. Results

### 2.1. Characterization of Doap1 Gene Structure

Comparison between the genomic and cDNA sequences revealed that *doap1* gene includes one internal intron. RNA-seq data (partially published [25,26]) supported the transcribed region with 1977 bp (Supplementary Figure S1). TATA-box candidate sequences were found upstream of the transcribed region, indicating the presence of a promoter for *doap1* gene. It would be reasonable to consider that translation of DOAP1 starts from the most forward start codon in the RNA-seq supporting region, thus the start codon was predicted to locate 93 bp-downstream from the transcription initiation site (Supplementary Figure S1).

The coding region is estimated to produce a polypeptide with 562 amino acid residues (~59.0 kDa, Supplementary Figure S2). Sequence features of DOAP1 were examined with the SignalP [27] and InterProScan algorisms, and it was predicted that DOAP1 contains an *N*-terminal signal sequence (ranging from M1 to A19), as well as quinonprotein alcohol dehydrogenase-like superfamily (IPR011047, ranging from Q18 to P184). Subcellular localization of DOAP1 was predicted with a series of bioinformatics tools we have utilized [17,25], and as a result ER-localization was assumed. A proline knot motif was found in the plant oleosome-associated proteins, and demonstrated to work as a specific signal for oleosome-targeting in plants (oleosins) [28,29]. In the case of microalgal oleosome-associated proteins found to date, the proline knot motif is not present, but proline-rich hydrophobic domain is contained [19]. Similarly, DOAP1 is less likely to have the proline knot-like motif, while a proline-rich region (Supplementary Figure S2) and a highly hydrophobic region [17] individually exist at the *C*-terminus. BLAST screening revealed that *Phaeodactylum tricornutum*, a pennate diatom same with *F. solaris*, is the only organism which has an ortholog of this protein according to the present national center for biotechnology information (NCBI) database, while its function remains unknown.

### 2.2. GFP Expression in the Transformants

**Figure 1:**
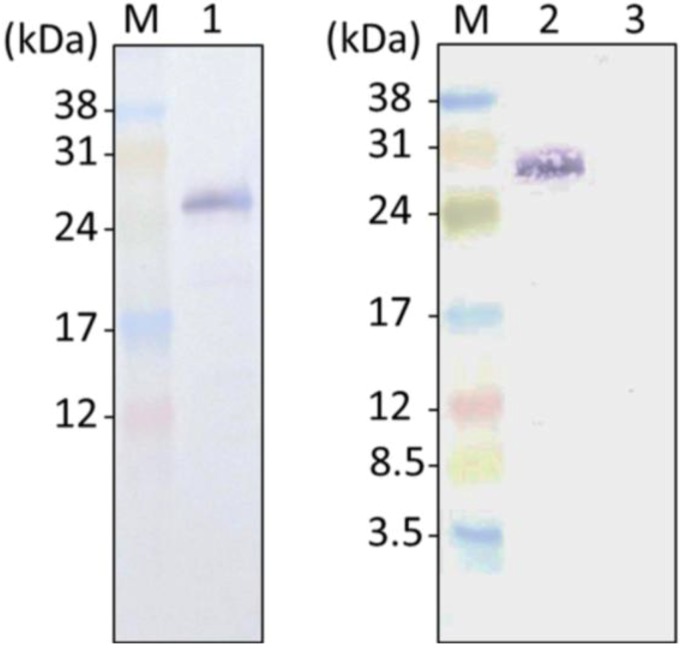
Green fluorescence protein (GFP) detection with Western blotting from *F. solaris* JPCC DA0580 transformants expressing neat GFP (**Lane 1**); S_DOAP1_-GFP (**Lane 2**); and wild-type cells (**Lane 3**). Lane M represents molecular marker.

To confirm the initial subcellular localization of DOAP1, we attempted to express the fusion protein of DOAP1 signal sequence and GFP (S_DOAP1_-GFP). Although the DOAP1-coding region was assumed as mentioned above, we determined to fuse the DNA fragment ranging from the tentative promoter region to the predicted coding region of the *N*-terminal 57 amino acid of DOAP1 (including the signal sequence and a part of mature DOAP1, see also Supplementary Figures S1 and S2) with *green fluorescence protein* (*gfp*) gene in order to ensure the actual native signal sequence can be expressed. It should be noted that our previous study also utilized the same DNA region to express the full length of DOAP1 fused with GFP [17]. The constructed expression vector was introduced into *F. solaris* cells. As-prepared transformants were subjected to Western blotting to confirm whether the fusion protein S_DOAP1_-GFP was produced in the cells. A specific band was visualized in the transformant sample using anti-GFP antibody (Figure 1). The detected protein was larger than the neat GFP produced in the *F. solaris* transformants (Supplementary Figure S3), suggesting the successful expression of the target fusion protein. Furthermore, its size was smaller than the intact protein corded (approximately 33 kDa); this could be caused by the cleavage of the signal peptide after transportation [30]. As a negative control experiment, wild type cells were also examined, and no signal was detected.

### 2.3. ER-Targeting of S_DOAP1_-GFP

**Figure 2:**
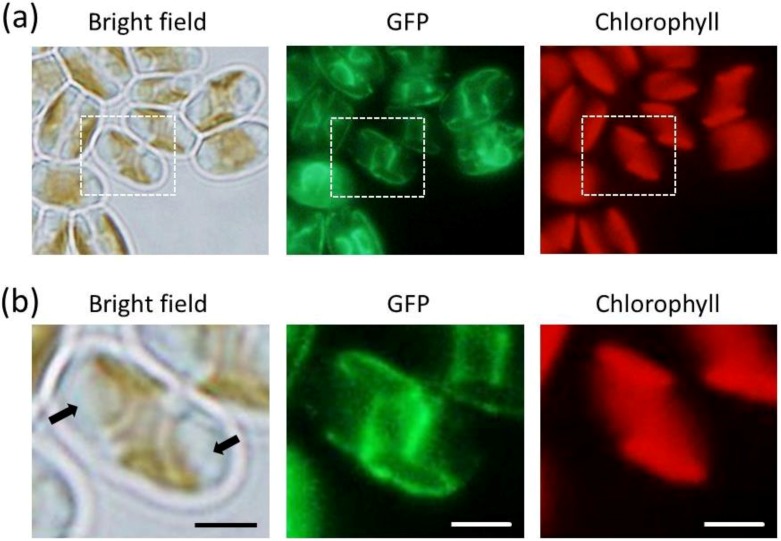
Microscopy studies on *F. solaris* JPCC DA0580 transformants expressing S_DOAP1_-GFP. (**a**) Bright field and fluorescent images of the transformants; (**b**) Magnified images of the square regions in (**a**). Black arrows represent oleosomes. (scale bar = 2 μm).

**Figure 3:**
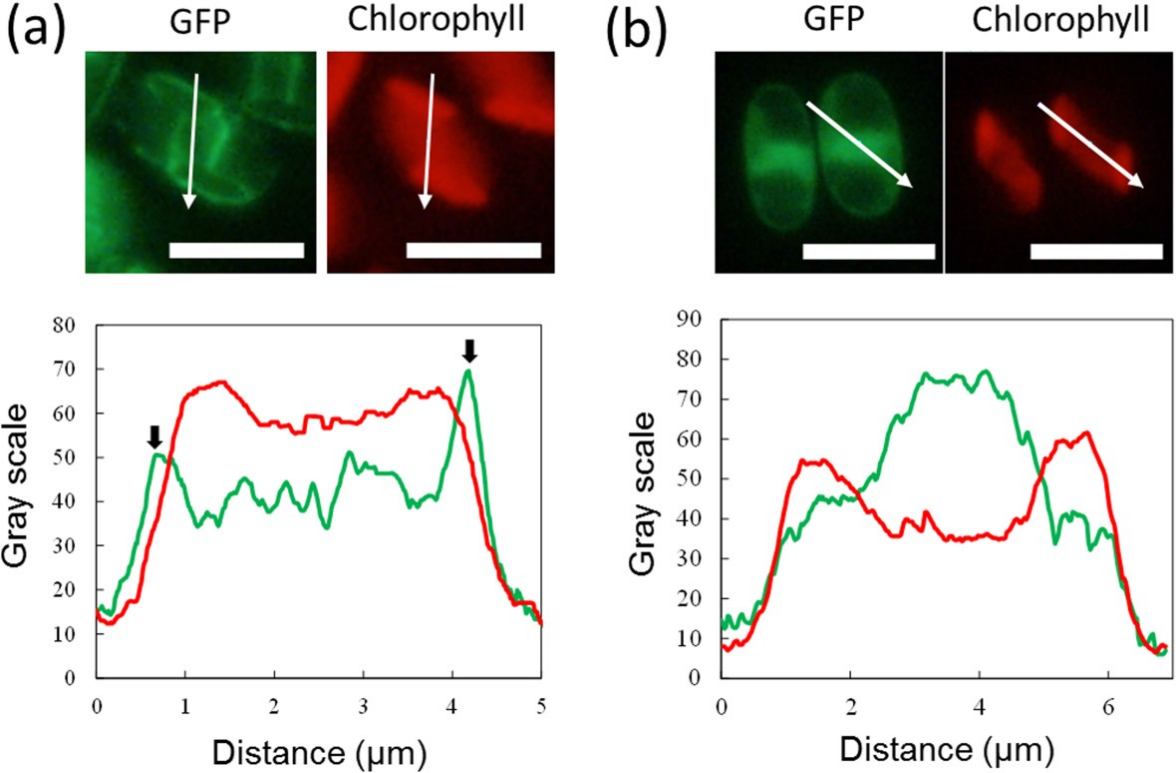
Fluorescence profiling on the microscopic images of *F. solaris* JPCC DA0580 expressing S_DOAP1_-GFP (**a**) and neat GFP (**b**). Fluorescent signals of GFP and chlorophyll along the white arrows in the images are shown in green and red lines, respectively. Fluorescent peaks of GFP outside of the chlorophyll fluorescent region are shown with the black arrows. (scale bar = 5 μm).

To examine whether the DOAP1 signal sequence directs proteins to specific organelles, the cells expressing S_DOAP1_-GFP were observed using a fluorescent microscope. The intense fluorescence was observed around the chloroplast, as well as central cellular region (Figure 2). This fluorescence distribution was obviously different from that of the fusion protein consisted of full length of DOAP1 and GFP, which strictly localized at the oleosomes [17]. When GFP and chlorophyll distribution was spatially profiled, it was demonstrated that the peak of GFP fluorescence was outside of the chlorophyll (Figure 3a). This feature was reproducibly confirmed in several cells (Supplementary Figure S4), suggesting that S_DOAP1_-GFP localizes outside the chloroplast. In the case of the transformants expressing the neat GFP, only the central cellular region emitted significant fluorescence, suggesting the expression at the cytoplasm (Figure 3b). When the cells were stained with the Hoechst dye, the nucleus was demonstrated to localize at the center of the cell, and surrounded by the GFP emission (Supplementary Figure S5).

**Figure 4:**
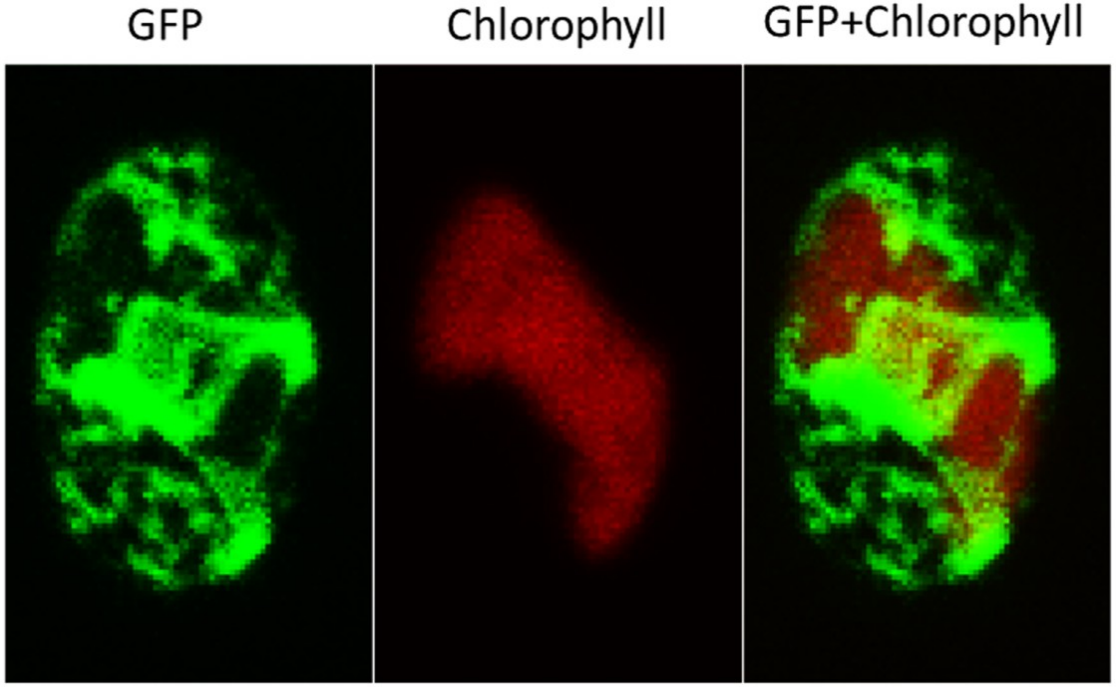
Confocal microscopic images of *F. solaris* JPCC DA0580 transformant expressing S_DOAP1_-GFP.

Confocal microscopy reconstituted three-dimensional (3D) arrangement of S_DOAP1_-GFP in the cells. GFP fluorescence delineated the network of membranes thought to represent the ER (Figure 4 and Supplementary Figure S6). The fluorescent network extended through the cell, and surrounded the chloroplast. This 3D arrangement closely resembles that observed in *P*. *tricornutum* expressing GFP at the ER [24]. When *F. solaris* was stained with an ER-specific dye, a similar feature was observed (Supplementary Figure S7).

## 3. Discussion

DOAP1 has been identified an oleosome-associated protein through our proteomic research. It was predicted to have 506 amino acids in length previously [17]. However, recently obtained RNA-seq data allowed us to find the *N*-terminal extension, which we have fortunately introduced in the expression vector in the previous study [17] but did not consider that this extra sequence may be a part of the coding region. By SignalP algorism, this sequence is predicted to be an ER-targeting signal sequence which can be recognized by a Sec61 complex for transition across the ER membrane [31].

Next, to test whether this tentative signal sequence can route proteins into the ER of *F. solaris*, we constructed the expression vector of S_DOAP1_-GFP, where the signal sequence and a part of mature DOAP1 (M1–M57 in the Supplementary Figure S2) were together fused at the *N*-terminus of GFP. Western blotting for the *F. solaris* transformants harboring the S_DOAP1_-GFP expression gene confirmed substantial expression of the target protein, as well as the cleavage of the signal sequence. Although the cleavage site is likely to be the point between A19 and Q20 according to the SignalP algorism, actual cleavage site still remains unclear, and can be elucidated with *N*-terminal sequencing of the cleaved protein [24]. *In vivo* localization of S_DOAP1_-GFP in *F. solaris* was observed with fluorescent microscopy. As a result, intensive green fluorescence was detected surrounding the chloroplast and nucleus (Figure 2 and Figure 3, and Supplementary Figure S4 and S5). Confocal microscopic analysis further revealed the meshwork-like structure showing GFP fluorescence around the chloroplast, suggesting that the target fusion protein was transported into the ER. It has been widely accepted that diatoms have complex chloroplasts which are surrounded by four membranes owing to their evolutional history with secondary symbiosis [32]. The innermost and second innermost membranes are actually corresponding to the two membranes of the primary chloroplast. The space between the second outermost membrane and the outermost membrane represents the ER (also known as chloroplast-ER; CER). Organelle-specific targeting by recombinant proteins in diatoms has been studied in a pennate diatom, *P*. *tricornutum* [24,30,33,34]. ER-targeting has been achieved by fusing the specific signal presequence at the *N*-terminus of proteins, while the inner chloroplast-targeting needed an extra transit sequence-like domain following the ER signal. In the case of ER-targeting by GFP, meshwork of green fluorescence was observed around the chloroplast as well as the nucleus in *P*. *tricornutum* [24,30]; the GFP distribution was similar with that observed in this study. These previous studies also support that the GFP distribution in this study represented the ER-targeting of S_DOAP1_-GFP. ER-staining with the specific dye further supported this notion.

When DOAP1 with its full length was labeled with GFP, fluorescence was only observed on the oleosomes [17]. In contrast, the truncated DOAP1 including the signal sequence and the following partial mature protein sequence (Supplementary Figure S2) directed the GFP into the ER, and little fluorescence was observed from the oleosomes (usually two oleosomes exist in a *F. solaris* cell at the polar position). These results suggest that DOAP1 could localize in the ER at first due to the *N*-terminal signal sequence, then it is transported onto the oleosomes. Specific signals for targeting oleosome (e.g., proline knot) is not yet identified in DOAP1, thus the transportation mechanism from the ER to oleosomes still remains unknown. We assume that the proline-rich region at the *C*-terminus of DOAP1 might be a possible candidate of oleosome-targeting signal because similar feature was found in lipid droplet surface protein (LDSP) in other microalga, *Nannochloropsis* sp. [19]. Incidentally SignalP did not detect ER-signal sequence from LDSP. In order to specify the oleosome-targeting sequence in DOAP1, the GFP fusion experiment with various truncated forms of DOAP1 will be performed in the near future. The protein transportation from the ER to oleosomes also implies the direct interaction between these two organelles, otherwise DOAP1 cannot eventually move to the oleosomes. This notion is consistent with our previous study, in which the oleosome membrane was hypothesized to be derived from the ER membrane [17].

Another significance of this study is establishment of the method for ER-specific recombinant protein expression in the oleaginous microalga. At the ER, many critical reactions with regard to the biofuel productivity and quality take place. For instance, acyl-chain desaturation and elongation would be performed at ER [25], and the acyl-chain length and desaturation degree directly affects the resultant biodiesel fuel quality [35]. Engineering these metabolic pathways by transporting heterogeneous enzymes could be a promising approach to control the fuel quality derived from *F. solaris*, and the DOAP1 signal sequence could achieve the transportation by just fusing it at the *N*-terminus of the target proteins.

## 4. Experimental Section

### 4.1. Culture Conditions

The marine diatom, *F. solaris* JPCC DA0580, was isolated from the junction of the Sumiyo and Yakugachi Rivers, in Kagoshima, Japan (28°15'N, 129°24'E) [21]. *F. solaris* was cultured in the f/2 medium [36] (75 mg NaNO_3_, 6 mg Na_2_HPO_4_·2H_2_O, 0.5 μg vitamin B12, 0.5 μg biotin, 100 μg Thiamine HCl, 10 mg Na_2_SiO_3_·9H_2_O, 4.4 mg Na_2_-EDTA, 3.16 mg FeCl_3_·6H_2_O, 12 μg CoSO_4_·5H_2_O, 21 μg ZnSO_4_·7H_2_O, 0.18 mg MnCl_2_·4H_2_O, 70 μg CuSO_4_·5H_2_O, and 7 μg Na_2_MoO_4_·2H_2_O) dissolved per liter of artificial seawater. Transformant cells were incubated in the f/2 medium with antibiotics G418 (500 μg/mL). Cultures were aerated with sterile air at 25°C under 140 μmol/m^2^/s of continuous illumination.

### 4.2. Characterization of Nucleotide and Protein Sequences

DNA sequence neighboring the tentative *doap1* (*g12504*) gene was retrieved from our domestic database of *F. solaris* whole genome sequence (Supplementary Figure S1) [37]. cDNA sequence and RNA-seq data regarding *doap1* gene has already been obtained in our previous studies [17,25]. TATA-boxes upstream of *doap1* gene were predicted with the polymerase II promoter function of GENETYX ver.10. Protein features were analyzed with SignalP [27], InterProScan and BLAST.

### 4.3. Vector Construction and Transformation

The expression vector, pSP-DOAP1GFP/GAPDH, for full length of DOAP1 (formerly g4301) fused with GFP, was constructed in our previous study [17], in which the predicted coding region (without intron) and its up-stream sequence (638 bp) were synthesized (Integrated DNA Technologies, Inc., Coraville, IA, USA), and inserted between the glyceraldehyde 3-phosphate dehydrogenase (GAPDH) promoter derived from *F. solaris* and *gfp* gene. Transcription is terminated by the fucoxanthin chlorophyll a/c-binding protein A (fcpA) terminator derived from *P. tricornutum* [38]. In this study, the DNA fragment including a part of *doap1* gene was amplified by polymerase chain reaction (PCR) using the primer pair (5′-ATGTTCCCTGGGCATTCGTG-3′ and 5′-CTTGTCTCCCGACAACAAGATG-3′) and pSP-DOAP1GFP/GAPDH as a template. The amplified fragment was inserted between the same promoter and GFP gene. The constructed plasmid was referred to as pSP-S_DOAP1_GFP/GAPDH.

Transformation of *F. solaris* was performed by microparticle bombardment using the Biolistic PDS-1000/He Particle Delivery System (Bio-Rad Laboratories, Inc., Hercules, CA, USA) as described previously [17,38].

### 4.4. Western Blotting

*F. solaris* transformants (1 × 10^7^ cells) were collected by centrifugation, washed with water, suspended in 100 μL of 1% (*w*/*v*) sodium dodecyl sulfate (SDS) in aqueous solution and boiled for 10 min. After centrifugation, supernatant was collected, and SDS sample buffer was added (final concentration of 62.5 mM Tris-HCl, pH 6.8, 5% 2-mercaptoethanol, 2% SDS, 5% sucrose, and 0.002% bromophenol blue). Denatured proteins were separated by SDS-polyacrylamide gel electrophoresis (SDS-PAGE) using a 12.5% (*w*/*v*) gel, and transferred to a polyvinylidene difluoride membrane. GFP was then detected using alkaline phosphatase (ALP)-labeled anti-GFP antibody (Rockland immunochemicals Inc., Gilbertsville, PA, USA, 1/5000 dilution from stock in PBS containing 0.05% Tween 20). BCIP/NBT-Blue (Sigma, St. Louis, MO, USA) was used as the ALP substrate for visualization.

### 4.5. Fluorescent Microscopy and Image Analysis

Transformant cells were observed using a fluorescent microscope BX51 (Olympus Corporation, Tokyo, Japan); a NIBA filter set for GFP, a WIG filter set for chlorophyll and a WU filter set for Hoechst 33342 fluorescence, respectively. Confocal microscopy was performed with Fluoview FV1000 (Olympus Corporation, Tokyo, Japan). Hoechst staining was conducted by adding Hoechst 33342 (Invitrogen, Eugene, OR, USA) to the cell culture at 1:50 volume ratio (final concentration = 200 μg/mL). The images obtained were analyzed with Image J program. In order to display the grayscale of the GFP and chlorophyll fluorescence, the fluorescence images obtained were converted into 8-bit black-and-white images, and then plot profiling was performed.

## 5. Conclusions

The fusion experiment with GFP and the signal sequence of the oleosome-associated protein DOAP1 revealed that DOAP1 contains the signal sequence targeting the ER. This result suggests that DOAP1 could initially be transported into the ER with the aid of its signal sequence, and subsequently transported onto the oleosomes. This new finding implies the interaction between the ER membrane and oleosome. Protein targeting to the ER achieved in this study is also useful for engineering the lipid synthesis pathway in *F. solaris* because key reactions for lipid synthesis including elongation and desaturation of acyl chains occur in the ER. This could contribute to improvement of biodiesel quality derived from *F. solaris*.

## Supplementary Files

Supplementary File 1 Supplementary Information (PDF, 786 KB)
