# Supplementary material for: Oleosome-Associated Protein of the Oleaginous Diatom Fistulifera solaris Contains an Endoplasmic Reticulum-Targeting Signal Sequence
Source: Mar Drugs. 2014 Jun 30;12(7):3892–903. doi: 10.3390/md12073892 (PMC4113804; doi:10.3390/md12073892)
Supplement: Supplementary File 1 — Supplementary Information (PDF, 786 KB) [file marinedrugs-12-03892-s001.pdf]

Supplementary Information

Figure S1. DNA sequence neighboring *doap1* gene (*g12504*) on the *Fistulfera* sp. genome. DOAP1-cording region predicted in this study is shown in green. An intron in *doap1* gene is shown in blue. TATA-boxes predicted with GENETIX ver.10 are shown with rectangular boxes. The region supported by RNA-seq data ranges from the closed triangle to the open triangle. The region fused with GFP gene in this study is shown in a red box.

```
                                                   50                                                   100
ATGTTCCCTG GGCATTCGTG TGTGGTCCTT CGCGCACGCT TCGTGGACTG CCGAAAGCGT GGACTTACAC GCGGCGGGAC TGATGGGTGG CACGTAAGTA
                                                  150                                                   200
TCCAAATCCA ACCCCACAGT TGAGTATGGA GCCAGGTATA CTCCCATCCG TTTCATTCAT GGACTAAGAG TAGGCCAACG TCCGTTGTCA ACTAACTATA
                                                  250                                                   300
CTAAATAATG TTCTAAGCGT TGCCGGAGTG GATTGTACAA AAATGGATCG TGGGAATATT CTTTACGTCG GCGCGGAAGA AATATTTAGA AGCACCGGAC
                                                  350                                                   400
CGTTTCAACA TCCGTTCAAC CGTACACTCG ATCGATGACG AAAGTGTGCA ATACATCGTG TTTTTCTCCC TCCAAAGATT CACATTTCGA CGGACGAAAC
                                                  450                                                   500
GATTAACACA CAATACGACT TGGTGGACGT TGCAAAGTAT TTGATTCGTA TTATTTATTT GTTTGCAACC ATGATGAAAC TCtCCTTGAG CTTTCTTCTT
                                                  550                                                   600
CTCTTCTTGA GCTCCCTCGT TCAGGCTCAA GAGTTCAACT ACCTCGATCC GACGGTGTTG TGGTCGGGAC GAACGACCGA TGACACGGCC GCCGGAGTCT
                                                  650                                                   700
TGCAGGGCAA CGGTATCTAC TTGTCTCCCG ACAACAAGAT GATTGTTTCC ACATCATCGG ATGGTACTTT GCGAGCCTTT GAACCAACGA CGGGTGCCGT
                                                  750                                                   800
CTTGTGGACT TTTGTCCCCG AAAATCTCGG TTTTGCCATT CGTTGTTGGA GTGGTGTCAC TTTTAACTAC CGCAGCGCGC GTCCTTATTT GGTTTATGCG
                                                  850                                                   900
ATTGCGGATG GTGCTACCGA CAAGACGAAC AGTCCCTTGG CGACGACGTG AGTACTTTTG TTGATGATAA TGCTGACGTC TTTCCTTTTC TCACTCCTCT
                                                  950                                                  1000
TTTCTCGACA GTCGTGTGAT TGCCGTCGAC CCGACGGGTG CTCTCATCTT CCAATCGGCT TCCTTGCAAG GTATTGCTGC CGGAACTCCC GTCACGGACA
                                                 1050                                                  1100
GTGACGGAGG TCACATTCTC GTTACACACA ATGTGGATGC TCTGGAAGGA TACTTGTCCA TCTTTTCGGT GGCCGGCTTG CAACCCGGTG CTCCCCTCGA
                                                 1150                                                  1200
ACCCATCTTT TCCGAAAACT ATTCGTCGCA AGAAAGCGGT TCCAACGGTA CCGTCGCCGT GGCCCGTCCC TTTTCTCCCA TTGGTTTCTA CCACAATCCC
                                                 1250                                                  1300
ACGGCCGGTT GGTACGACGG CGGTGCCAAC AACACCAACG ACGTCTTCTT CTTTGCGTGG GATACTCCCC GCACAGCCAC ATCGATTGCC GAAACGGACG
                                                 1350                                                  1400
GTCAAGTCTT TGTCTTTCAA TTCCCCATGG AGTACATGGG AGACGGCCAG GGACTCGGAT TCGCTCCCAT GGGCAGTCGT ACCGACTTTC ACACCACCAC
                                                 1450                                                  1500
CGGCCCCGTC TTGACCAACG GAGGTCTCAG CATGTACTGG ACCGTCTCCA AGGCTACCAC CGACTGCTAC CTCGGAGAAG CCCCCTTGGC CCGTACGTAC
                                                 1550                                                  1600
TTTTCGCGTG GCCGCACCGG TCGTGCCACC TTTGATCGCG CCGATCGACC CGCTCCGGCC TACCAAGGAC CCCGTGCCCA AGTCACCCTC AGCAGTGATC
                                                 1650                                                  1700
CCGTCCAACC GATGGTGTAC GGTGTCGGTG CCGCTCCCCA AATCTGGTCC ATGTCGTACG ATTACAGCAA CATGAATGTC GCCACCACTC CCGATCTCGT
                                                 1750                                                  1800
GTCGTCGCGT CTCTTGTTGA CGCAAGATCA GCAATACATT ATTTATGCCA CACAAGCGAC CGTTTCGACG GATGGTGGAG TTCACATGGT CCCCGTCGGC
                                                 1850                                                  1900
AACTTGGGAC AGTTGGACTG GACCGTCAAT GTCACGGGTG GTGTCTATGG TGATATCGCC TTGAATGGAC GTGGTACCAT TGTGTATGCG GCCGATGTAT
                                                 1950                                                  2000
TGGGACAGAT TTCGGCGTAC CAGTTTGGTG AGAACTTTGC CGCGACACCG GCCCCGACCT TGTCTCCCAC TTCGGACAGT ACGTTGGCTC CAACATCGGG
                                                 2050                                                  2100
TGATGATGGT GAATCGGATG TCCCCACTCC GGCCTTTGGT ACGATTGTGC CGACCCCCTC GGGCAGTGGA CCCGAAGACG GTGAATCTCC CGCTCCCAGT
                                                 2150                                                  2200
GCTCCGGATG GTACGACCGT TCCTCCCGCC ACGGGACCCA GTGTTCCTCC TCCCGTCGAT TCGTCCTCGG CGAACATGGC CGTAGTGGGT ATGGCATCCA
                                                 2250                                                  2300
TGTTGTTGGC ATTGGTTCTT TAAATCATGT CGCCGCACAA TGGCTAAATG GATAGTAGAT ACGATATAAT ATTGGATGCT AGAAGAGTCG TACGATAGAA
                                                 2350                                                  2400
TTAACTAGAT GTTTGCTCGA AAAAGTGCGT GTGTATGCTA AACCGCCGGG GAAATAGTGA TTTCTCCAAG ACTCGTGGTA ATAGACAATT TTTCGTCTTT
                                                 2450                                                  2500
GTTCAATGCA TGAATCTAGT ATTGCGCGCG GGAGTCGTAC AATCGAATCA AATCGTTGCT GATTGGAAGA TGCATATGTC ATTGATGAGT AGTAGAAAGA
                                                 2550                                                  2600
CGAATAGTGG AAAAGCCCAC GAACCATGAC TCTGTTCGGC AGGACTAGTA CGACTCTTGG TGGTTTCGTC TCCTTCCAGC TCTACTTGAC TGGAAGTATC

CGACCACAGA CTATCGTTGT TC
```

Figure S2. Predicted amino acid sequence of DOAP1. The signal sequence predicted with the SignalP program is shown in orange. Quinonprotein alcohol dehydrogenase-like domain (IPR011047) is shown in blue. The region fused with GFP gene in this study is shown in a red box.

```
        10         20         30         40         50
MMKLSLSFLL LFLSSLVQAQ EFNYLDPTVL WSGRTTDDTA AGVLQGNGIY

        60         70         80         90        100
LSPDNKMIVS TSSDGTLRAF EPTTGAVLWT FVPENLGFAI RCWSGVTFNY

       110        120        130        140        150
RSARPYLVYA IADGATDKTN SPLATTRVIA VDPTGALIFQ SASLQGIAAG

       160        170        180        190        200
TPVTDSDGGH ILVTHNVDAL EGYLSIFSVA GLQPGAPLEP IFSENYSSQE

       210        220        230        240        250
SGSNGTVAVA RPFSPIGFYH NPTAGWYDGG ANNTNDVFFF AWDTPRTATS

       260        270        280        290        300
IAETDGQVFV FQFPMEYMGD GQGLGFAPMG SRTDFHTTTG PVLTNGGLSM

       310        320        330        340        350
YWTVSKATTD CYLGEAPLAR TYFSRGRTGR ATFDRADRPA PAYQGPRAQV

       360        370        380        390        400
TLSSDPVQPM VYGVGAAPQI WSMSYDYSNM NVATTPDLVS SRLLLTQDQQ

       410        420        430        440        450
YIIYATQATV STDGGVHMVP VGNLGQLDWT VNVTGGVYGD IALNGRGTIV

       460        470        480        490        500
YAADVLGQIS AYQFGENFAA TPAPTLSPTS DSTLAPTSGD DGESDVPTPA

       510        520        530        540        550
FGTIVPTPSG SGPEDGESPA PSAPDGTTVP PATGPSVPPP VDSSSANMAV

       560
VGMASMLLAL VL
```

Figure S3. Western blotting signals derived from GFP (**a**) and S_{DOAP1}-GFP (**b**) were plotted with red lines. Molecular marker signals at the neighboring lanes were also plotted with blue lines. For better comparison between (**a**) and (**b**), each marker position is marked with gray column.

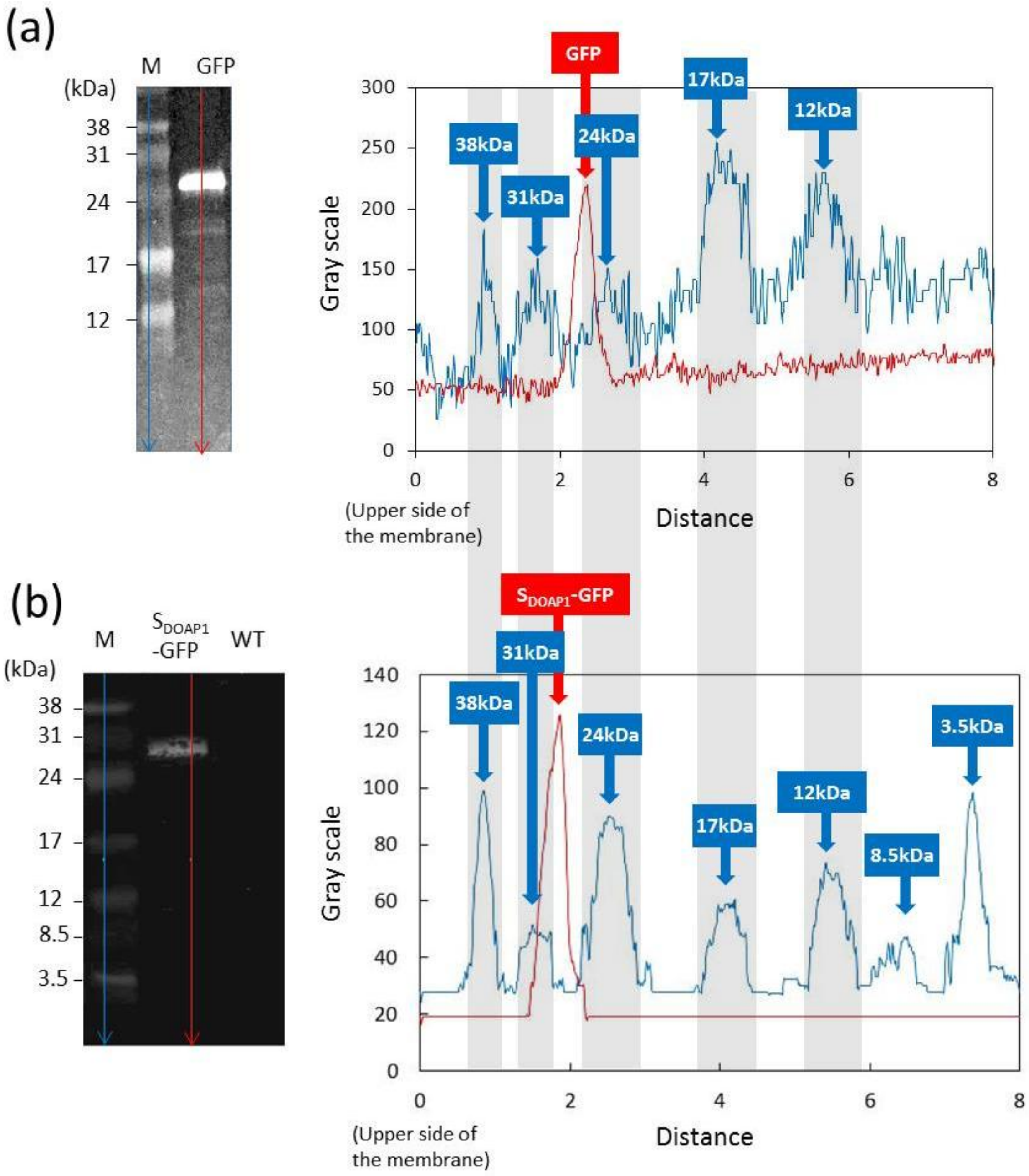

Figure S4. Examples of fluorescence profiling on the microscopic images of *Fistulifera* sp. JPCC DA0580 expressing S_{DOAP1}-GFP. Fluorescent signals of GFP and chlorophyll along the white arrows in the images are shown in green and red lines, respectively. Fluorescent peaks of GFP outside of the chlorophyll fluorescent region are shown with tje black arrows. (scale bar = 5 μm).

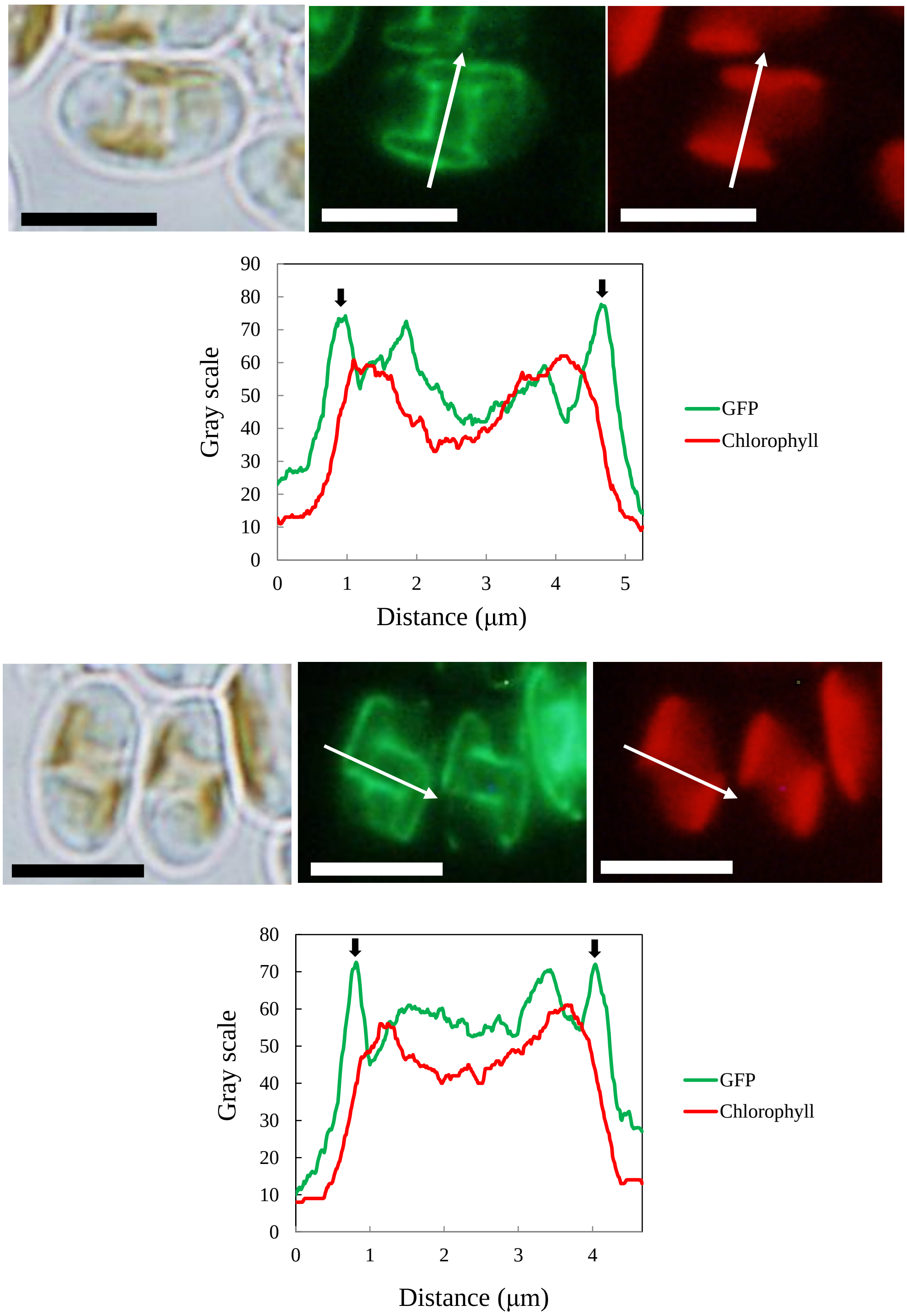

Figure S5. Microscopic observation on *Fistulifera* sp. JPCC DA0580 transformants expressing SDOAP1-GFP with Hoechst 33342 staining. Hoechst staining was conducted by adding Hoechst 33342 (Invitrogen, Eugene, OR, USA) to the cell culture at 1:50 volume ratio (final concentration = 200 µg/mL) (scale bar = 5 µm).

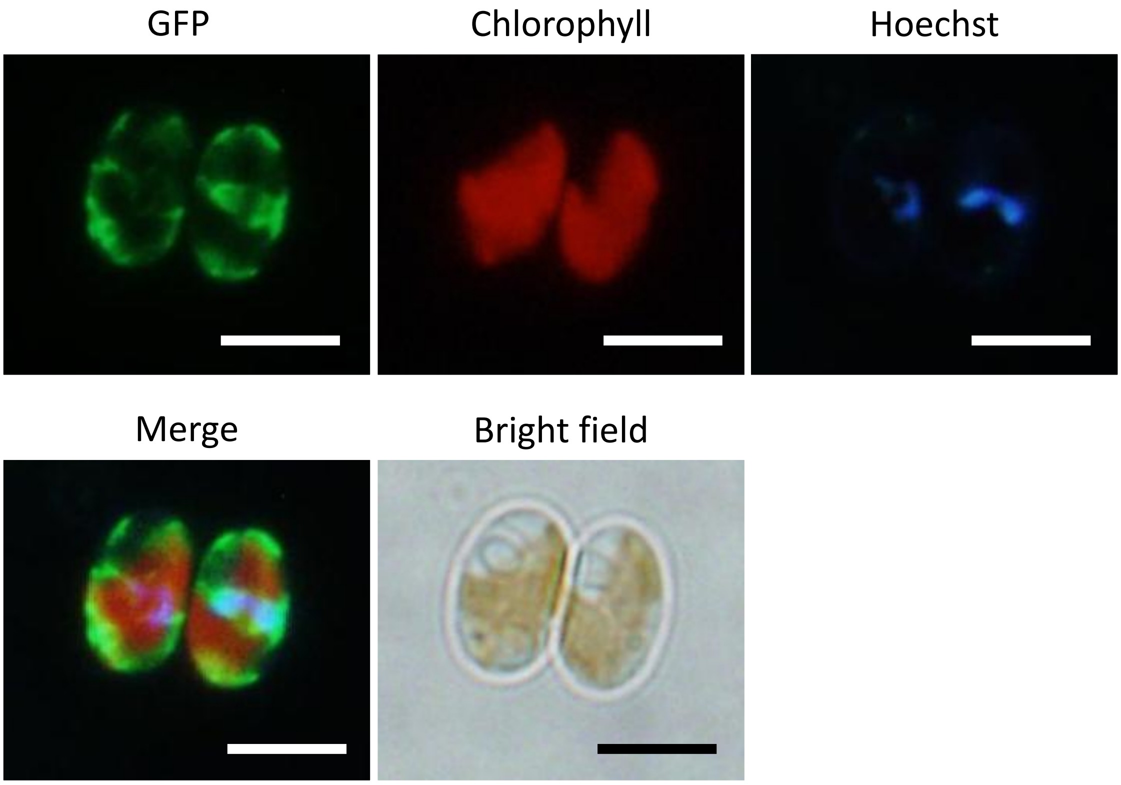

Figure S6. X-Y plane sequence images generated from the reconstructed 3D-images of Figure 3 in the main text (scale bar = 1.3 µm).

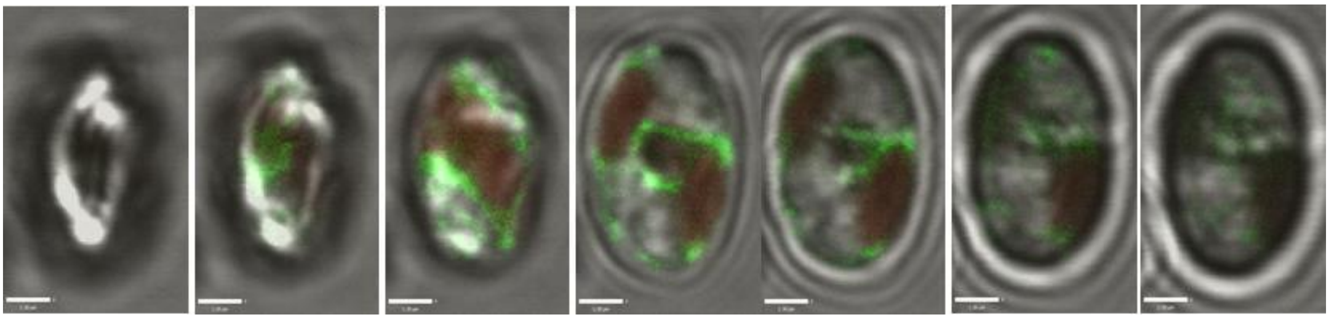

Figure S7. Confocal microscopic images of Fistulifera sp. JPCC DA0580 stained with ER-Tracker™ Green (Life Technologies). The cells (1 × 107 cells/mL) were incubated with 0.78 µg/mL of ER-Tracker™ Green in phosphate buffered saline for 30 min.

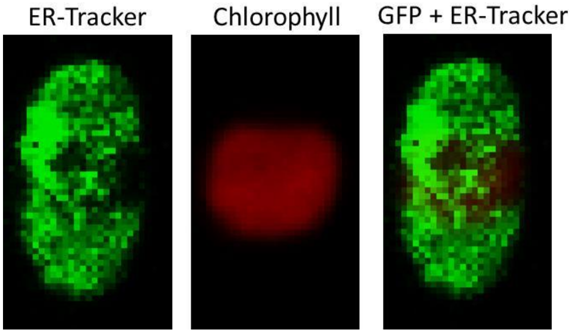

© 2014 by the authors; licensee MDPI, Basel, Switzerland. This article is an open access article distributed under the terms and conditions of the Creative Commons Attribution license (http://creativecommons.org/licenses/by/3.0/).
